# Postictal psychosis: presymptomatic risk factors and the need for further investigation of genetics and pharmacotherapy

**DOI:** 10.1186/1744-859X-5-9

**Published:** 2006-07-21

**Authors:** Eric M Morrow, Jennifer M Lafayette, Edward B Bromfield, Gregory Fricchione

**Affiliations:** 1 Department of Psychiatry, Massachusetts General Hospital, Boston, MA 02114, USA; 2 Division of Epilepsy and EEG, Department of Neurology, Brigham and Women's Hospital, 75 Francis Street, Boston, MA 02115, USA

## Abstract

**Background:**

Postictal psychosis (PIP), an episode of psychosis occurring after a cluster of seizures, is common and may be associated with profound morbidity, including chronic psychosis. Symptoms are often pleomorphic, involving a range of psychotic symptoms, including hallucinations and disorders of thought. PIP is treatable and may be averted if presymptomatic risk factors are considered in susceptible patients and treatment is initiated.

**Case presentation:**

In this report, we present an illustrative case of PIP. The patient, Mr. R, presented to our emergency room with delusions and disordered thought process following a cluster of seizures. He recovered after admission, sedation and treatment with antipsychotic medication.

**Discussion:**

A list of presymptomatic risk factors is established based on review of current literature. Identification of such risk factors may potentially help with prophylactic treatment; however, little empirical research exists in this area and treatment guidelines are thus far largely based on expert opinion. Further, while the neurobiology of schizophrenia is advancing at a rapid pace, largely due to advances in genetics, the pathophysiology of PIP remains largely unknown. Considering the progress in schizophrenia research in the context of the clinical features of PIP and existing studies, potential neurobiological mechanisms for PIP are herein proposed, and further genetic analyses, which may help identify those susceptible, are warranted.

**Conclusion:**

While PIP is an important problem that may present first to general hospital psychiatrists, as in the case presented, this topic is under-represented in the medical psychiatry literature. As discussed in this article, further research is needed to develop presymptomatic screens and treatment pathways to help prevent morbidity.

## Background

Postictal psychosis (PIP) is characterized by an episode of psychosis occurring within one week after a cluster of seizures. PIP is common. In a study of inpatient video-electroencephalographic monitoring, the annual incidence of postictal psychotic events was estimated to be 6.4% [1]. The prevalence of PIP is difficult to measure; however, in a study of greater than 100 outpatients with treatment resistant partial epilepsy, the prevalence of having experienced postictal psychotic symptoms was found to be 7% [2]. While PIP is usually short-lived, with remission after several days to weeks, chronic psychoses may develop from recurrent episodes or even a single episode [3]. Psychopathologic symptoms across cases are pleomorphic [1,4]. Psychotic symptoms may include hallucinations, including auditory or visual. Abnormalities of content of thought, such as ideas of reference or delusions (including paranoid, grandiose, somatic, religious or others) are often present, as may be abnormalities of form of thought (for example, thought blockage, loose associations or tangentiality). Schneiderian First-rank symptoms have been reported [1]. In a minority of cases, manic symptoms may co-occur in the acute episode after the seizure cluster, yet these symptoms generally are not of sufficient duration to meet criteria for manic episode, while the psychotic symptoms more commonly persist [1,4]. Given this prevalence and morbidity, assessing risk for PIP (or prompt recognition of symptoms) in patients who have had a cluster of seizures may have major therapeutic implications. Expert opinions suggest that episodes may be averted or shortened by introducing low-dose neuroleptics at the first sign of psychosis or sleeplessness, which often precedes episodes; however, little empirical evidence exists in the literature on this topic. In some situations, the consultation psychiatrist, or as in the case below, the emergency room psychiatrist, are faced with the task of recognizing and treating this condition. Despite the fact that PIP represents 25% of the psychoses of epilepsy (POE) [5,6], it is relatively underrepresented in the psychiatric medicine literature. Here we describe a case which is remarkable for the extent to which it demonstrates the classic features of PIP, and it thereby may serve to review the diagnostic criteria, known risk factors, and treatment supported by experts. Recent insight into the genetics of primary psychotic disorders such as schizophrenia may lead hypotheses into the susceptibility and pathophysiology of PIP which is also discussed herein. However, overall this article is designed to point out the need for further research in both pathophysiology and treatment of this common condition.

## Case presentation

Mr. R, a 47 year-old right-handed male, presented to our hospital, confused, with delusions of grandeur and elevated mood. He was initially found wandering our hospital lobby at night somewhat bewildered and lethargic. Left in the front drive of the hospital by a taxi cab, the patient said that he had a "seizure attack." He was carrying multiple bags as if he were on a trip. Shortly after being taken to the Emergency Department (ED), Mr. R became agitated, at which time he received intramuscular haloperidol and lorazepam, and intravenous phenytoin. The patient slept and an EEG was performed.

Information was gathered by telephone contact with the patient's family, and we determined that Mr. R was married and lived in a different city where he was stably employed. The patient had a 4 year history of generalized tonic-clonic (GTC) seizures, perhaps secondarily generalized, and thought to be related to a motor vehicle accident with head trauma and loss of consciousness 11 years prior. He also had a history of febrile convulsions as a child, but there was no known history of CNS infection or developmental delay. There was no family history of seizures.

According to the patient's wife, Mr. R had a cluster of four witnessed convulsive seizures four days prior to presentation. Behaviour noted prior to convulsions included "staring, not answering, chewing, swallowing heavily, moving both hands in a swimming motion or picking at things." Following the four witnessed seizures, three days prior to presentation in our hospital, the patient was brought to his local hospital by his wife where he was admitted. Valproic acid was added to the patient's anti-epileptic regimen of oxcarbazepine. He had an approximately 24 hour period of normal behaviour and was discharged. Two days prior to presentation at our hospital, Mr. R had one sleepless night. His wife described the patient's behaviour as "hyper" and "strange". He then left his home unannounced, taking belongings with him. In the 24 hours prior to presenting at our hospital, Mr. R made numerous telephone calls to family and friends around the country, making it evident that he was traveling. His reported statements contained delusions of grandeur and wealth, and reflected impulsivity and poor judgments. This behaviour represented a drastic departure from the Mr. R's baseline according to his wife.

Other than the seizure disorder as stated above, past medical history was non-contributory. He had no prior psychiatric history. Substance and alcohol use were not significant. There was a notable family history of depression without suicidality. There were no known environmental exposures. Mr. R was raised as a Christian, but he did not participate in religious activities as an adult.

In the ED, the patient's vital signs, physical exam, cranial nerve, motor and sensory examinations were all within normal limits. On mental status examination after 12 hours in hospital, Mr. R was in his undergarments and a hospital gown, sitting without an appropriate sense of modesty. He was reading a copy of the Old Testament. He was friendly, co-operative and jovial. His speech was somewhat rapid but interruptible. He reported his mood as "really good. Everyone thinks something is wrong when you're really happy." His affect was bright and at times even euphoric. His thought process was tangential with loose associations, disorganized and at times difficult to follow. His thought content was notable for delusions of grandeur and wealth with religious content, but not of a bizarre nature. Hallucinations were denied and not suspected by behaviour on exam. Mr. R described a sense that he knew the examining clinician, stating "Are you sure I haven't met you before?" His language was fluent with normal naming and repetition. Cognitive exam was notable for difficulty with tasks of attention. Review of physical symptoms was notable for denial of macropsia or micropsia, gustatory or olfactory hallucinations, or stomach upset at the time of exam or at any time in the course of his recent illness by report.

Laboratory values revealed normal basic chemistries including liver function tests, coagulation tests, and CBC. Urine and extended serum toxicology tests were negative. Valproic acid level was 21 micrograms/milliliters. Serum creatine kinase levels rose to 1830 U/L at 16 hours after presentation then fell towards normal, with normal troponin levels. Serum prolactin levels were elevated to 20.4 ng/mL at 9 hours after presentation. Ammonia, vitamin B12, serum folate, and thryroid measures were normal, and RPR was non-reactive. ESR, ANA, and rheumatoid factor were within normal limits. Hepatitis C and B and HIV serology were negative. Chest x-ray was normal. A lumbar puncture was not performed.

Brain CT was within normal limits except for mildly prominent ventricles. MRI of brain was notable for encephalomalacic change within bilateral posterior frontal and right parietal lobes, best seen on FLAIR sequences. No abnormal contrast enhancing or mass lesions were noted in the mesial temporal lobes. Multiple hyperintense foci in deep and subcortical white matter were noted on FLAIR bilaterally. Ventricles, sulci and cisterns were mildly prominent for age and there was mild cerebral cortical volume loss.

An electroencephalogram was obtained in the ED on presentation shortly after receiving phenytoin, lorazepam and haloperidol. Diffuse low amplitude slowing with frontocentral predominance was observed, in addition to central and symmetric spindles. No spikes or sharp waves were seen.

Mr. R was admitted to inpatient psychiatry and treated with antiepileptic and antipsychotic medicine. Oxcarbazepine was administered at a dose of 600 mg twice daily, as was valproic acid at a dose of 500 mg three times daily and 750 mg at night. Lorazepam was administered at 1 mg three times daily for 3 days, then tapered. Olanzapine was used at a final dose of 15 mg daily. Disorganized form of thought, delusional content, poor self care and inappropriately bright affect remained until hospital day 8 but began to improve at this time. A repeat EEG was performed on hospital day 5. Both drowsy and asleep EEGs were without definitive evidence of epileptiform activity. Brief periods of awake state showed an unsustained but symmetric 8–10 Hz posterior rhythm. The patient was discharged on hospital day twelve with significant improvement in his mental status.

## Discussion

Logsdail and Toone defined diagnostic criteria for PIP [4], thereby distinguishing PIP from the other psychoses of epilepsy, namely ictal and interictal psychosis. The patient presented here meets these diagnostic criteria, which include: 1) an episode of psychosis emerging within one week after the return of normal mental function following a seizure; 2) the episode length was between 24 hours and 3 months; 3) there was no evidence of anticonvulsant toxicity, a previous history of interictal psychosis, EEG evidence of nonconvulsive status epilepticus, recent head injury, or alcohol or drug intoxication. Though these criteria may seem clear after the fact, diagnosis in the ED can be difficult if the complete history is not known. Other life threatening disorders that may lead to a change in mental status such as acute intoxication or withdrawal, head injuries, intracranial bleeding, infection and metabolic abnormalities must be ruled out.

In terms of DSM-IV [7] diagnostic criteria, the case presented here meets criteria for a Psychotic Disorder with Delusions Due to a General Medical Condition, specifically epilepsy. In general, PIP is meant to be categorized under this diagnosis. The patient would not be classified as a Delirium as during the 8–10 day period during which his delusions persisted, he was without changes in consciousness. In addition, while the case presented here has some qualities of Dissociative Fugue, such as unexpected travel away from home, the patient was without amnesia or identity confusion, and this diagnosis is only applied in the absence of known medical causes. For this reason also, Brief Psychotic Disorder is excluded. Because the patient described here presented with manic symptoms in addition to psychotic symptoms, Mood Disorder with Manic Features Due to General Medical Condition is an important consideration. Because this patient had a sustained period (over one week) where delusions persisted in the absence of other prominent manic symptoms, we favour the diagnosis of Psychotic Disorder, over Mood Disorder due to General Medical Condition. Mood disorders, particularly depression, associated with epilepsy (and including in the postictal state [1]) are an important problem with profound morbidity; however, this topic is beyond the scope of this review and has been reviewed recently elsewhere [8].

Several studies, generally conducted in inpatient video-EEG monitoring units, have characterized risk factors for susceptibility to developing PIP. The patient presented here typifies many of these risk factors (Table 1). Patients who have had a cluster of seizures are at risk [9]. Many studies have suggested that patients with GTC seizures or partial seizures secondarily generalized are at increased risk [10,11]. While there is a variation in the age of onset of epilepsy, there is a trend toward later onset epilepsy and a duration of 5 to 10 years between the onset of chronic epilepsy and PIP [9,11]. Prior episodes of PIP are considered a risk factor, as are prior psychiatric hospital admissions [1,4,10,12]. PIP may be recurrent and some studies suggest that in select cases psychosis may not resolve entirely between episodes [3]. Family history of mood disorder (as in our case) has been implicated [13]. Family history of psychosis has been noted in one study [14]. However, in two studies psychosis was not statistically different between patients with and without family history [1,4]. Despite these equivocal data, given that these studies are small and that overwhelming evidence supports an elevated recurrence risk for primary psychotic disorders, we believe that family history of psychosis is a likely risk factor in addition to a family history of mood disorders. There is not strong evidence implicating gender. While some studies report more cases in men than in women [1,11], these studies are too small for statistical significance. A larger study is required prior to considering male gender as a strong, independent risk factor. An absence of history of febrile seizures has been reported in some studies [9] although this has not been found consistently [10]. A history of low intellectual function has been noted as a risk factor in some studies [14].

**Table 1:** Risk Factors for Postictal Psychosis

A cluster of seizures.
Insomnia within 1 week, but particularly within 1–3 days.
Epilepsy of > 10 years duration.
Generalized tonic-clonic seizures or complex partial secondarily generalized.
Prior episodes of PIP.
Prior psychiatric hospitalizations or history of psychosis.
Bilateral independent seizure foci (particularly temporal).
History of traumatic brain injury or encephalitis.
Low intellectual function.
In a patient with chronic epilepsy with the above risk factors, patients should be monitored vigilantly and low dose antipsychotic should be considered at the first signs of insomnia, or the earliest signs of psychosis or thought disorder. If patient has a history of recurrent PIP, antipsychotics may be considered prophylatically after seizure cluster.

Interestingly, bilateral cerebral abnormalities have been commonly reported in patients with PIP. Several studies have reported bilateral temporal independent discharges in patients with PIP [4,9,10,15]. In a careful study by Devinsky et al., patients with PIP had evidence of bilateral cerebral injury or dysfunction more frequently than control epilepsy patients [10]. There was a significantly greater rate of history of encephalitis or head trauma. Such patients often have bilateral frontal and temporal cerebral damage. This pattern of neuropathology was noted on MRI with the patient presented here. While EEG evidence of a seizure focus was not obtained in our patient, the lip-smacking and automatisms preceding convulsions, as described by the wife, are highly suggestive of temporal lobe seizures which become secondarily generalized. Interestingly, a SPECT study of patients with TLE and PIP was remarkable for bifrontal and bitemporal hyperperfusion patterns [16], suggesting the bilaterality may be an important pathological feature of PIP.

While no prospective trials of treatment, symptomatic or prophylactic, have been published, treatment recommendations have emerged based on expert opinion and have appeared in the neurology literature [1,6,15]. Agents and doses, therefore, have been generally chosen based on studies and experience with acute psychosis. Multiple authors encourage vigilant monitoring of patients with risk factors for PIP after a cluster of seizures. In such patients, low dose antipsychotic medication (2–4 mg of risperidone) is recommended in the early stages after the emergence of symptoms [6]. As patients are often neuroleptic-naïve, starting at low doses may be prudent and such doses are often sufficient. Most authors favour atypical antipsychotics; however, some literature describes using typicals, such as haloperidol (2–5 mg) with success [1]. Some authors even argue that pharmacotherapy may be instituted prior to the advent of psychosis, and instead with the emergence of sleeplessness which can portend a psychotic episode [6]. In patients who have chronic epilepsy and a history of recurrent PIP, Kanner has suggested that treatment may be considered after the cluster of seizures alone [6]. Patient and family education may be critical if the seizure cluster occurs in the outpatient setting. Although no studies have been performed specifically to assess longitudinal treatment guidelines, experts and the available literature recommend that patients should not remain on antipsychotic medication. Instead the medicine may be prescribed for 2 to 5 days if the episode is averted, or up to 6 months if a full-blown episode occurs, as in our patient, and then may be tapered. Because antipsychotics may lower the seizure threshold, it may be necessary to increase doses of antiepileptic medicines. Again, readers should be cautioned that prospective trials assessing these treatment strategies have not yet emerged in the literature. One small case series, for example, suggests that sedation, as opposed to specifically antipsychotic treatment, is sufficient to ward off PIP; however, this study is underpowered and retrospective [17]. Further research specifically targeting treatment and prevention of PIP is important, as some data suggest that PIP may be prevented and conversion to chronic psychosis may be at risk [3]. For example, in this case, could Mr. R's episode of psychosis have been averted? Potentially yes, if he had been administered a dose of neuroleptic during the episode of sleeplessness which preceded his psychosis.

The pathophysiologic mechanism of PIP is unknown. One hypothesis is that PIP represents a psychic Todd's paralysis [15,18]. In one of the few studies on the pathophysiology of PIP, Fong et al. observed lateral temporal hyperperfusion as assessed by SPECT in postictal psychosis [19], and they thereby argue against the Todd's paralysis hypothesis which might predict hypoperfusion. The temporal emergence of PIP after a lucid period following seizures may also be inconsistent with the decrescendo course of Todd's phenomena [20]. Therefore, a role for Todd's phenomena remains controversial, yet given our limited knowledge of the etiology of psychosis, a component of a Todd's phenomenon may not be entirely excluded.

By considering the generally common clinical features of PIP, and calling on studies of the pathophysiology of schizophrenia, speculative models of the neurobiology of PIP may be constructed, and thereby tested in future studies. In schizophrenia, it is widely believed that mesolimbic dopamine input to the nucleus accumbens, as well as the amygdala and hippocampus, is related to the acute, positive symptoms of psychosis, and that this is the site of action of antipsychotic medicine (see [21] and references therein). Further, a cortico-hippocampal loop may be disrupted which results in excess subcortical dopamine. Neuroimaging studies widely support hypofunction (generally bilateral) of the frontal cortex in schizophrenia, particularly in the region of the dorsolateral prefrontal cortex [22], while hyperactivity, has been noted in the hippocampus [23]. One may speculate that in PIP, there exists bilateral hypofunction of the frontal and/or other multimodal association cortex. The fact that PIP is seen commonly in cases of bilateral dysfunction suggests this, as well as the loss of cortical inhibition of structures such as hippocampus and accumbens. At least in macaque, prefrontal cortical projections have been observed to project bilaterally [24,25], thereby, unilateral lesions would not be sufficient for loss of cortical inhibition. Further, this model would suppose that the cluster of generalized seizures commonly seen in advance of an episode of PIP would uncover the deficits of bilateral cortical lesions seen in head trauma or diffuse cortical damage seen post-encephalitis. For example, in the MRI in the current case, bilateral lesions were observed in regions of frontal cortex likely to be multimodal association cortex (see case above). These lesions are consistent with head trauma known from the history to precede the emergence of the seizure disorder. In addition, generalized cerebral cortical volume loss was noted which could contribute to further generalized loss of cortical inhibition. Also as in schizophrenia, hyperfunction of the temporal cortex (more specifically hippocampus) is likely. This follows from the observation that the majority of PIP occurs in patients with TLE [4,9,10,15].

In order to prevent cases of PIP and reduce morbidity, further research is warranted. A recent investigation of interictal psychosis has found an increase in psychotic symptoms in patients with low serum folate and/or high homocysteine levels [26]. In addition, given the recent emergence of putative schizophrenia susceptibility genes, genetic risk may be examined (reviewed in [27]). For example, the val158met polymorphism in the COMT gene, which is associated with prefrontal dysfunction in schizophrenia [28], could be assessed for a possible role in psychosis in patients with epilepsy. In addition to COMT, several other putative risk haplotypes have emerged in the last 5 years including in genes such as DISC1 and Neuregulin [27]. As the field of schizophrenia genetics works to make these susceptibility loci more definitive, they may also be tested for a potential role in predisposing to PIP. Such studies could draw the field of epilepsy further into the convergence of other neuroscientific areas related to psychosis, and in the future provide further clinical criteria for assessing risk.

## Conclusion

Postictal psychosis (PIP) is common and may be associated with profound morbidity. PIP occurs after a cluster of seizures and often after an episode of insomnia. Risk factors include longstanding generalized tonic-clonic seizures or complex partial seizures that are secondarily generalized. A prior episode of PIP is a strong risk factor, as well as prior psychiatric history, in particular a history of psychosis. Bilateral temporal seizure foci are common in PIP, as is a history of traumatic brain injury, encephalitis or low intellectual function (which may reflect bilateral cortical compromise). This condition may be averted with prudent treatment of susceptible patients with prophylactic antipsychotic and/or sedative medicines. Therefore, psychiatrists should be cognizant of risk factors and vigilant when evaluating susceptible patients.

## Competing interests

The author(s) declare that they have no competing interests.

## Authors' contributions

EMM, GF and EBB reviewed the existing literature. EMM drafted the manuscript which was edited GF, EBB, and JML. JML contributed to the thoughtful care of the patient presented, reviewed the manuscript and contributed to the writing. All authors approved the final manuscript.

## References

[B1] Kanner AM, Stagno S, Kotagal P, Morris HH (1996). Postictal psychiatric events during prolonged video-electroencephalographic monitoring studies. Arch Neurol.

[B2] Kanner AM, Soto A, Gross-Kanner H (2004). Prevalence and clinical characteristics of postictal psychiatric symptoms in partial epilepsy. Neurology.

[B3] Tarulli A, Devinsky O, Alper K (2001). Progression of postictal to interictal psychosis. Epilepsia.

[B4] Logsdail SJ, Toone BK (1988). Post-ictal psychoses. A clinical and phenomenological description. Br J Psychiatry.

[B5] Dongier S (1959). Statistical study of clinical and electroencephalographic manifestations of 536 psychotic episodes occurring in 516 epileptics between clinical seizures. Epilepsia.

[B6] Kanner AM (2000). Psychosis of Epilepsy: A Neurologist's Perspective. Epilepsy Behav.

[B7] American Psychiatric Association., American Psychiatric Association. Task Force on DSM-IV (2000). Diagnostic and statistical manual of mental disorders : DSM-IV-TR.

[B8] Kanner AM (2005). Depression in epilepsy: a neurobiologic perspective. Epilepsy Curr.

[B9] Umbricht D, Degreef G, Barr WB, Lieberman JA, Pollack S, Schaul N (1995). Postictal and chronic psychoses in patients with temporal lobe epilepsy. Am J Psychiatry.

[B10] Devinsky O, Abramson H, Alper K, FitzGerald LS, Perrine K, Calderon J, Luciano D (1995). Postictal psychosis: a case control series of 20 patients and 150 controls. Epilepsy Res.

[B11] Liu HC, Chen CH, Yeh IJ, Sung SM (2001). Characteristics of postictal psychosis in a psychiatric center. Psychiatry Clin Neurosci.

[B12] Adachi N, Kato M, Sekimoto M, Ichikawa I, Akanuma N, Uesugi H, Matsuda H, Ishida S, Onuma T (2003). Recurrent postictal psychosis after remission of interictal psychosis: further evidence of bimodal psychosis. Epilepsia.

[B13] Alper K, Devinsky O, Westbrook L, Luciano D, Pacia S, Perrine K, Vazquez B (2001). Premorbid psychiatric risk factors for postictal psychosis. J Neuropsychiatry Clin Neurosci.

[B14] Adachi N, Matsuura M, Hara T, Oana Y, Okubo Y, Kato M, Onuma T (2002). Psychoses and epilepsy: are interictal and postictal psychoses distinct clinical entities?. Epilepsia.

[B15] Savard G, Andermann F, Olivier A, Remillard GM (1991). Postictal psychosis after partial complex seizures: a multiple case study. Epilepsia.

[B16] Leutmezer F, Podreka I, Asenbaum S, Pietrzyk U, Lucht H, Back C, Benda N, Baumgartner C (2003). Postictal psychosis in temporal lobe epilepsy. Epilepsia.

[B17] Lancman MECW, Asconapé JJ, Penry JK (1994). Clinical management of recurrent postictal psychosis. J Epilepsy.

[B18] Fong GC, Fong KY, Mak W, Tsang KL, Chan KH, Cheung RT, Ho SL (2000). Postictal psychosis related regional cerebral hyperfusion. J Neurol Neurosurg Psychiatry.

[B19] Fong GC, Ho WY, Tsoi TH, Fong KY, Ho SL (2002). Lateral temporal hyperperfusion in postictal psychosis assessed by 99mTc-HMPAO SPECT. Neuroimage.

[B20] Boylan LS (2001). Postictal psychosis related regional cerebral hyperfusion. J Neurol Neurosurg Psychiatry.

[B21] Svensson TH (2000). Dysfunctional brain dopamine systems induced by psychotomimetic NMDA-receptor antagonists and the effects of antipsychotic drugs. Brain Res Brain Res Rev.

[B22] Weinberger DR, Egan MF, Bertolino A, Callicott JH, Mattay VS, Lipska BK, Berman KF, Goldberg TE (2001). Prefrontal neurons and the genetics of schizophrenia. Biol Psychiatry.

[B23] Heckers S (2001). Neuroimaging studies of the hippocampus in schizophrenia. Hippocampus.

[B24] McGuire PK, Bates JF, Goldman-Rakic PS (1991). Interhemispheric integration: I. Symmetry and convergence of the corticocortical connections of the left and the right principal sulcus (PS) and the left and the right supplementary motor area (SMA) in the rhesus monkey. Cereb Cortex.

[B25] Preuss TM, Goldman-Rakic PS (1987). Crossed corticothalamic and thalamocortical connections of macaque prefrontal cortex. J Comp Neurol.

[B26] Monji A, Yanagimoto K, Maekawa T, Sumida Y, Yamazaki K, Kojima K (2005). Plasma folate and homocysteine levels may be related to interictal "schizophrenia-like" psychosis in patients with epilepsy. J Clin Psychopharmacol.

[B27] Harrison PJ, Weinberger DR (2005). Schizophrenia genes, gene expression, and neuropathology: on the matter of their convergence. Mol Psychiatry.

[B28] Egan MF, Goldberg TE, Kolachana BS, Callicott JH, Mazzanti CM, Straub RE, Goldman D, Weinberger DR (2001). Effect of COMT Val108/158 Met genotype on frontal lobe function and risk for schizophrenia. Proc Natl Acad Sci USA.

